# State of the Art of Telecommunication Systems in Isolated and Constrained Areas

**DOI:** 10.3390/s21093073

**Published:** 2021-04-28

**Authors:** Laurent Ferrier, Hussein Ibrahim, Mohamad Issa, Adrian Ilinca

**Affiliations:** 1 Wind Energy Research Laboratory, Université du Québec à Rimouski, Rimouski, QC G5L 3A1, Canada; Adrian_Ilinca@uqar.ca; 2 Institut Technologique de la Maintenance Industrielle, Sept-Îles, QC G5L 3A1, Canada; Hussein.Ibrahim@itmi.ca; 3 Institut Maritime du Québec à Rimouski, Rimouski, QC G5L 3A1, Canada; missa@imq.qc.ca

**Keywords:** telecommunication towers, optical fiber, Loon balloon, Stratobus, captive balloon, link budget, drones, coverage of a telecommunication system

## Abstract

Smart objects are deployed globally, contributing to improved communications and the growth of industrial systems’ performances. Unfortunately, isolated territories are generally excluded from this progress. Remote areas in Canada are no exception. Thus, about two hundred thousand people are living in isolated regions in Canadian territory. The development of these communities is slowed down not only by an outdated energy supply, but they are also dependent on telecommunication systems not fully deployed in those regions, thus contributing to the amplification of those populations’ isolation. Furthermore, in many remote regions of the planet, radio communication remains difficult due to geographical constraints, environmental attenuation, and the lack of telecommunication infrastructure. These regions are often referred to as “white areas,” meaning zones with little or no communication coverage. As a part of this article, a state of the art of telecommunication solutions available in an isolated environment is applied with a critical analysis based on several criteria. It shows the ability to use an original approach based on a captive balloon. Despite the proposed solution’s feasibility, several challenges need to be addressed before formally adopting it. These challenges include: (i) controlling the height of the balloon; (ii) stabilization of the balloon; and (iii) powering the system. The list of references given at the end of the paper should offer aids for the industry and for researchers working in this field.

## 1. Introduction

Smart objects are being deployed globally, thus contributing to improved communications and the growth of industrial systems’ performances. In the urban environment almost everywhere, the technological challenges are slowly being lifted, and the advances in this sector are exponential. Unfortunately, isolated territories are generally excluded from this progress [1]. Indeed, most communities living in these regions are apart from the rest of the planet. Their geographical isolation is characterized by the absence of a road network and rough weather conditions, especially during the winter.

For example, in Northern Canada, temperatures can be very low in the winter: between −40 and −50 °C during long periods. Snowfall is also frequent, and the height of the snow mixed with ice is significant. In that context, the population’s movement conditions are more difficult, which reinforces individuals’ isolation. This geographical isolation, reinforced by the difficult climate, is aggravated by complex and too costly telecommunication solutions. The lack of technological development, including access to energy and communications, penalizes the population in isolated regions. They are generally more affected by poverty and have less equipment than the industrialized zones to deal, for example, with environmental problems.

We count about two billion people living in these isolated sectors around the world. Regions of Northern Canada are no exception. Thus, about two hundred thousand people are living in isolated regions in Canadian territory. Nunavut [2] is, in that regard, an excellent example of an isolated territory [3]: Inuit communities are not connected to the electricity network of the province despite the presence of very productive hydroelectric stations [4]. Even though the resources in clean, renewable energies [5] are abundant, the fourteen communities must use non-renewable fossil fuels. However, the development of those communities is not only slowed down by an outdated energy supply [6], they are also dependent on the fact that the telecommunication systems are not or not much deployed in those regions. Yet, an efficient telecommunication system allows, among other things, to connect people of the whole planet. As the information revolution accelerates, universal access to those new telecommunication technologies must federate the efforts to introduce sustainable development.

The absence of telecommunication systems penalizes not only internal communications but also external communication. For example, an individual connected to other community members can be rapidly taken care of in an emergency. This is the case, for example, if one’s physical integrity is threatened or if a violent environmental event occurs in the isolated region. The communities could be warned upstream, and an emergency plan could be put in place to avoid, or at least reduce human disasters. Thus, sensors could be placed in the isolated sector’s strategic places and be linked to create a network, generating critical warnings of changing nature.

Quebec’s North Shore, with the northern trains of the Tshiuetin company (“north wind” in Innu), is a concrete example of the contribution that a network of sensors in an isolated environment could provide. The company transports ore from Schefferville (north of Quebec) to Sept-Îles, and it also carries passengers. The company operates the section of track between Schefferville and Emeril. However, this isolated sector extends over approximately 250 km. A network of sensors connected through a powerful telecommunication system would make it possible to secure the journey by warning, for example, the driver of a fall of minerals on the track or locating a broken train in full winter [7]. The cost of responding to a disaster when an adequate telecommunication system could have avoided it is staggering. As proof, the derailment of the QNS & L train on 6 November 2014, following a landslide that the driver saw too late [8], is an example of an accident that could have been avoided. The train descended a slope to dive into the Moisie River (northeast of Sept-Îles), causing the driver’s death.

Another example is the event that occurred in 2013 when a company train broke down near Emeril camp in Labrador. On that day, the temperatures were, with the wind factor, around −50 °C. There was no more heating on the train, and the passengers, including young children, had to regroup for several hours in a wagon to fight the biting cold while waiting for help. Fortunately, Route 500 or Trans-Quebec-Labrador was not far away, which allowed their evacuation. It must be considered that if the failure had occurred upstream in the woods, some passengers probably would not have survived. A telecommunication system would likely have avoided this problem. The start of this railway line is the isolated town of Schefferville, which can only be reached by train or plane.

In addition to the previous observations, technological disconnection [9] and lack of energy are not the only causes of the accentuation of the geographic isolation of populations [10]. In fact, isolated territories are unfortunately subject to harsh climatic conditions, especially during the winter season. Winter temperatures can be very low: between −40 and −50 °C for long periods. Snowfall is also frequent and the depth of snow mixed with ice is significant. The conditions of movement of the population are, in this context, more difficult. The isolation of individuals is, therefore, reinforced. The harsh winter also adds a significant constraint on existing or likely-to-be-developed telecommunications devices. However, this technological challenge of electronic device operation under challenging climates also appears in very hot climate isolated regions. It is the case, for example, in the African deserts.

Another difficulty penalizes the establishment of telecommunication systems in isolated territories, thus contributing to the amplification of those populations’ isolation. In many remote regions of the planet, radio communication remains difficult due to geographical constraints, environmental attenuation, and the lack of telecommunication infrastructure. These regions are often referred to as “white areas [11],” meaning zones with little or no communication coverage [12].

The article’s objective is to make a state of the art of telecommunication systems suitable in isolated regions to highlight an original and effective solution adapted to this type of environment. Before proceeding further, the satellite connection, which has been used in isolated areas for many years at a high cost [13], will not be part of this study. Our contribution in this article is to review telecommunication solutions for isolated areas and present an original solution based on tethered balloons.

The paper is structured as follows: Section 2 presents the state of the art of telecommunication systems in an isolated and constrained environment. Section 3 compares communication technologies according to several criteria to emphasize the advantages and disadvantages (benefits and limitations), while Section 4 further describes the chosen solution. Finally, Section 5 concludes the paper with possible future directions of study in this area.

## 2. Telecommunication Systems in an Isolated and Constrained Environment

**Figure 1:**
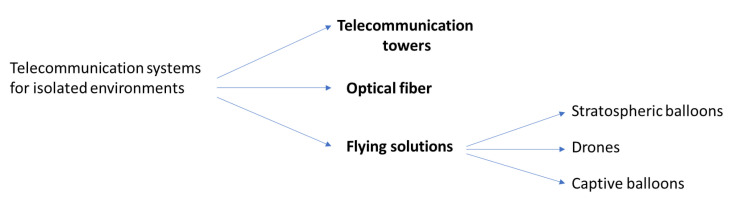
Telecommunication solutions for isolated environment.

Three solutions (Figure 1) stand out for setting up a telecommunication system in an isolated and constrained environment:

- Telecommunication towers
- Optical fiber
- Flying systems

### 2.1. Telecommunication Towers

The first telecommunication tower was undoubtedly the Italian Marconi device when he succeeded, in 1895, in transmitting a signal over several hundred meters in Bologna. In 1903, Gustave Ferrié developed an electrolytic detector more sensitive than the Branly’s coherer. Receiver sensitivity is a fundamental element when transmitting a signal. It is the minimum power that the receiver must receive to restore the transmitted information. The lower the sensitivity of the receiver, the longer the distance between the transmitter and the receiver. Gustave Ferrié, therefore, continued his experiments to gain even more distance. He needed a higher antenna [14]. The height of the emission source is an essential factor in designing a telecommunication system to avoid obstacles during the propagation of the electromagnetic wave and thus avoid the fading problems linked to the wave’s reflection.

Fortunately, Gustave Eiffel put his Eiffel Tower at his disposal. Gustave Ferrié naturally set up antennas on the latter. We certainly do not realize that the Eiffel Tower was promised to be destroyed after 1900 following the universal exhibition. However, it only owed its survival to being an extraordinary site for receiving transmitters and antennas. Unfortunately, for radio frequency specialists, it is not possible to find an Eiffel tower in every neighborhood. However, metal telecommunication towers (Figure 1) allow the transmitter and the receiver to gain height. The height of the transmitting and receiving antennas avoids problems of reflection of the signal transmitted on the ground (“ground reflection”) and of fading, meaning the reflection of the signal transmitted on objects (mobile or not). These two effects significantly reduce the range of the telecommunication system.

When designing a telecommunication system, engineers try to get as close as possible to a direct path for the wave transmitted to the receiver, a configuration called “fine of sight” or line of sight. Intermediate towers are installed between the one carrying the transmitter and the one containing the receiver to increase transmission distance. These towers act as relays by amplifying the signal strength and sometimes by changing the frequency. When these towers are built in an urban environment or close to the city, the electrical network supplies energy to onboard electronic devices. In contrast, in an isolated environment, energy is provided by micro-networks. These micro-networks generally use diesel generators. Hybrid systems were developed in recent years and introduced renewable energies with a preference for photovoltaic panels [15]. These solutions are viable when the sunshine potential of the site of installation allows it. These metal towers or pylons have a height that can vary between 30 m and 150 m. They can, in some cases, be guyed. In urban areas, buildings can serve as telecommunication towers.

**Figure 2:**
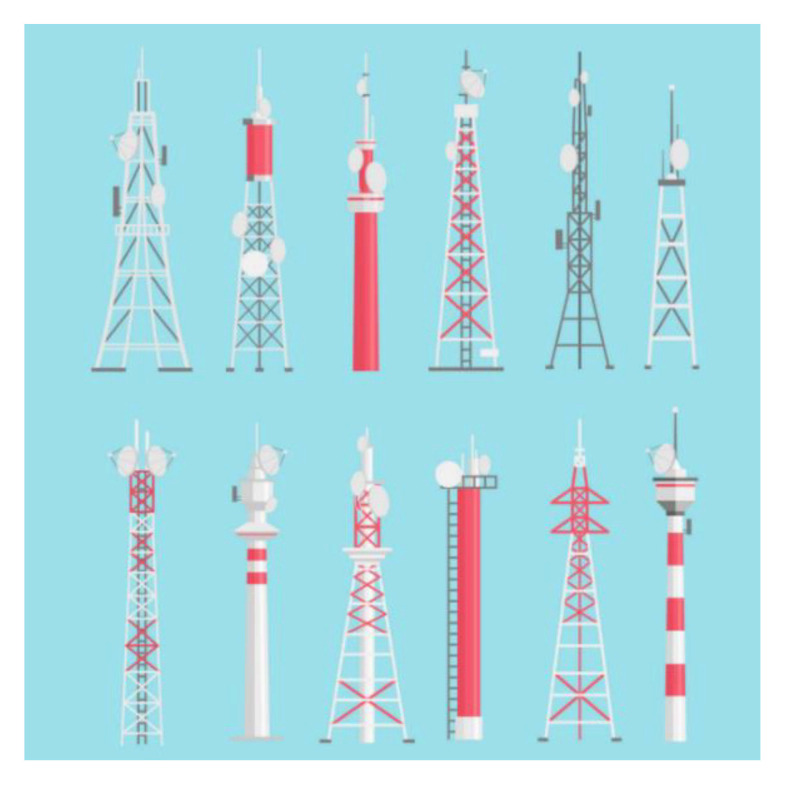
Different telecommunication towers set.

To correctly dimension a telecommunication system [16], we need to complete a link assessment. This encrypts the strength of the receiver’s signal located at a distance that we call from the transmitter. The chain of transmission appears in Figure 2.

The link budget [17] makes it possible to compute the strength of the signal received by the receiver (PR). We can extract the maximum distance between the transmitter and the receiver, and therefore the telecommunication system’s scope, by replacing the strength of the signal received by the receiver (PR) with its sensitivity. A receiver’s sensitivity is the minimum power received below which it cannot extract the information with certainty. The scope of the *Dmax* of the telecommunication system is:

(1) $$Dmax=\frac{\sqrt{\left(PT\times GT\times GR\right)\times \lambda^{2}}}{4\times \pi \times \sqrt{Sensitivity}}\;$$

where *Sensitivity* is the received power, *GT* is the antenna gain for the transmitter, *GR* is the antenna gain for the receiver, and *λ* the signal wavelength

Some important parameters appear in this expression in terms of telecommunications [18]. It is easy to see that the scope increases by increasing the signal strength at the transmitter level. Similarly, by increasing the carrier’s frequency, which conveys the information, the wavelength decreases and the scope declines. For example, by transmitting at the frequency of the UHF band, 915 MHz for the Internet of Things technology LoRa [19] (in America, 868 MHz in Europe), the transmitter and the receiver can be more distant than by transmitting by Wi-Fi at 5 GHz with equivalent transmitter power. However, another critical premise in telecommunications is that the data rate is lower when the frequency diminishes. Thus, with LoRa modulation [20], we are limited to low speed, 50 kbits/s. On the other hand, with 5 GHz Wi-Fi, we can easily transmit images with data rates of up to 2 Gbits/s. The Internet of Things for connected sensor-type objects requires the transfer of relatively small information for the moment. This bit rate of 50 kbits/s is, therefore, quite sufficient [21].

### 2.2. Optical Fiber

**Figure 3:**
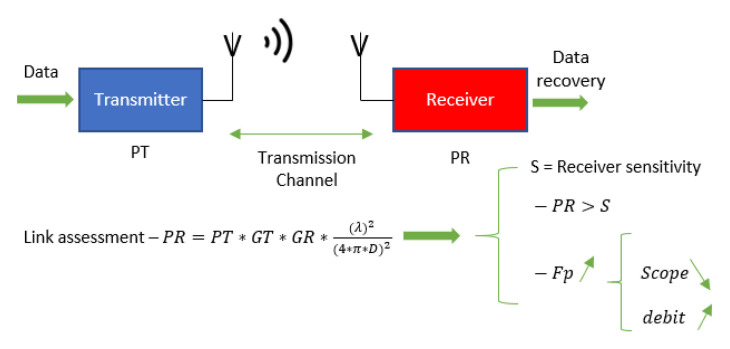
Link budget.

The other solution to consider when talking about setting up a telecommunication station to create a network of sensors [22] with the possibility of remote data access by an internet gateway is to use optical fiber [23]. For Quebec’s specific case, the North Plan was put in place by the government to reduce the digital divide by providing internet access in remote locations. Nunavik [24] is an example of this penetration of optical fiber (Figure 3) in the territory. The Cree indigenous communities have benefited from this development of optical fiber since 2011 for an amount of 29 million CAD. The second phase, which is about to be completed, will have cost 25 million CAD to deploy 800 km of optical fiber. Other communities will be connected to the Internet via optical fiber passing through undersea cables, even if the costs of deploying this solution are very high.

From a physical point of view, the optical fiber can be likened to a glass or plastic wire with the property of propagating light. Since light speed is 300,000 km/s, optical fiber makes it possible to obtain information rates significantly higher than those of coaxial cables that propagate the electrical signal at only 200,000 km/s with additional losses (weakening along the cable and coupling) [25]. Optical fiber has the following advantages:

- Lower attenuation than conventional cables propagating an electrical signal,
- Higher information rate,
- High propagation speed,
- High immunity to parasites, and
- Almost zero crosstalk.

One of the significant drawbacks of this type of technology is its fragility, which must be added to a higher cost. There are two main types of optical fiber, the multimode, for which there are different modes of light propagation within the fiber core, and the single-mode, in which there is only one mode of propagation of the light, the straight line way. In multimode optical fibers [26], there are two types of fiber, the step-index and the gradient index. The step-index optical fiber has a very large core with the disadvantage of a strong attenuation in the order of 10 dB/km. Its range is around 2 km and its speed around 100 Mbits/s. It is mainly used in local area networks (LAN). Multimode optical fiber with gradient index is also used in local area networks. Its core is of intermediate size (50 to 100 μm). It has better mitigation than that of step-index fiber. Its range is around 2 km and its speed around 1 Gbit/s. Monomodal fiber is the most efficient currently. This type of fiber is used in the core of global networks because its attenuation is almost zero (around 0.5 dB/km), its range in the order of 100 km, and its high speed of 100 Gbits/s [27]. It is evident that with the advent and development of this type of support, operators have embarked on a race to transmit more and more information through an optical fiber. To channel multiple sources of information over a medium such as optical fiber, the technique consists in using multiplexing. There are two types of multiplexing:

- Time division multiplexing (TDM)
- Wavelength division multiplexing (WDM)

TDM consists of cutting the optical fiber bandwidth in time intervals, which shares the different sources of communication. This allows multiple low-speed digital channels to be transmitted over a high-speed medium (optical fiber). WDM uses the mixing of several optical signals of different wavelengths on the same optical fiber. The wavelengths of the various sources are relatively distant to limit interference between them. We distinguish among the WDM, Coarse WDM, Dense WDM, and Ultra-Dense WDM [28]. Their respective performances are summarized in Table 1.

**Figure 4:**
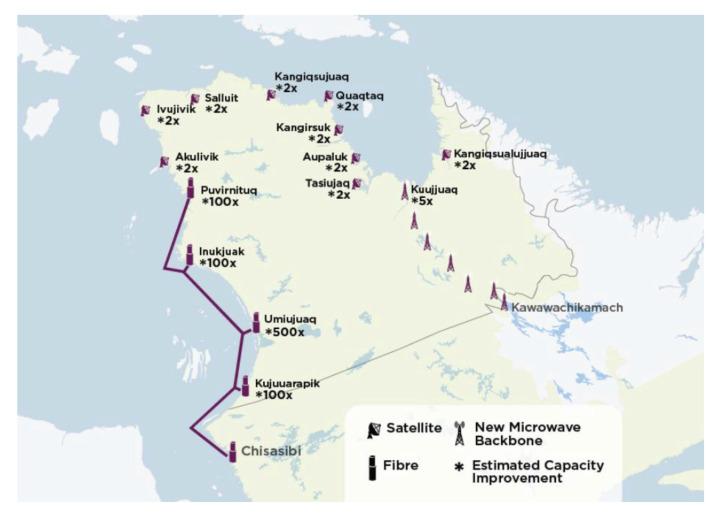
Nunavik’s optical fiber network.

It should be noted that the structural diagram method would need a multiplexer at the input and a demultiplexer at the output, as illustrated in Figure 4. The deployment of optical fibers over hundreds of kilometers involves relays (optical amplifiers) to amplify light [29], as shown in Figure 4.

### 2.3. The Flying Systems

The third solution, consisting of flying systems, can be separated into two items:

- The balloons, and
- The drones.

#### 2.3.1. The Balloons

**Figure 5:**
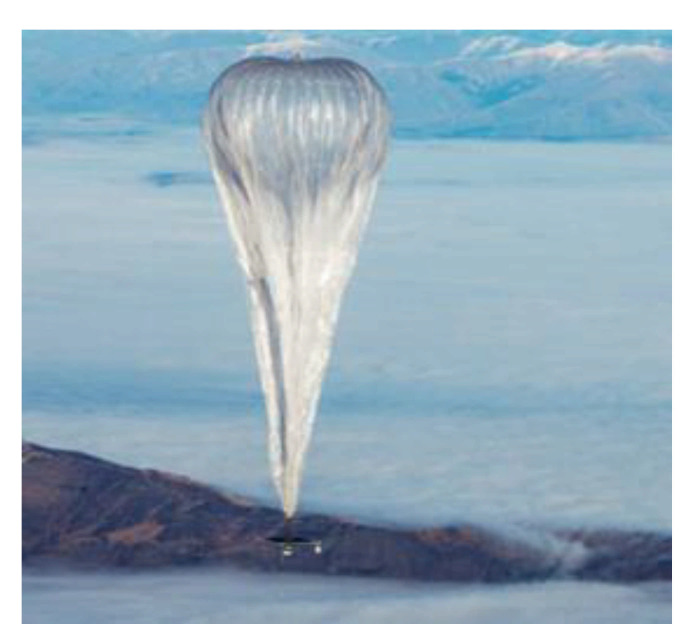
The Loon balloon [35].

The “balloon” solution [30] consists of future projects such as the Loon balloon [31] or the Stratobus. The Loon balloon (Figure 5) is a project by Google X, a subsidiary of Google. In 2013, in a context where 2/3 of the world population has no Internet access or very low-speed access, Google decided to create a long-term ring of connectivity around the world [32] to bring Internet access to the most remote areas of the globe. The solution adopted was to use helium-inflated balloons [33], which carried LTE antennas [34] ensuring 3G/4G speed and a rigid photovoltaic panel. These balloons take off from the ground and climb up to the stratosphere at 20,000 m, above the airliners’ altitude. They move according to the winds and are controlled and piloted from a ground control room. Thus, it is possible to converge several balloons in a sector and, therefore, ensure a covered area from a network point of view of the order of 1200 square kilometers.

This solution’s effectiveness was demonstrated during the tragic event of the hurricane in Puerto Rico [36] in October 2017. There were no longer any connections and people were in grave danger. Google deployed its balloons and local phone operators collaborated. This enabled many distressed people to find a hint of enough connectivity to send an SMS or an email alert. They were thus able to be located and saved. The payload that can be loaded is around 40 kg. Before the deployment in Puerto Rico, proof of autonomy had been carried out through a 187-day flight, with difficult conditions, as the balloon suffered winds of 220 km/h.

Thirty balloons were launched in 2013 from the southern hemisphere and New Zealand. Google is continuing its deployment with extensions in countries in the southern hemisphere such as Chile, Uruguay, and South Africa. This project is under development. One cannot connect of one’s own will. There will probably be agreements forged with telephone operators, but the costs are not known yet. Therefore, this solution will not be available to everyone soon, and it will probably not be suitable for all situations. It can also be expected that the cost of connecting intelligent objects or sensors to the autonomous telecommunication station in an isolated environment will probably be high. Thus, this type of balloon is not a solution for the autonomous telecommunication station.

**Figure 6:**
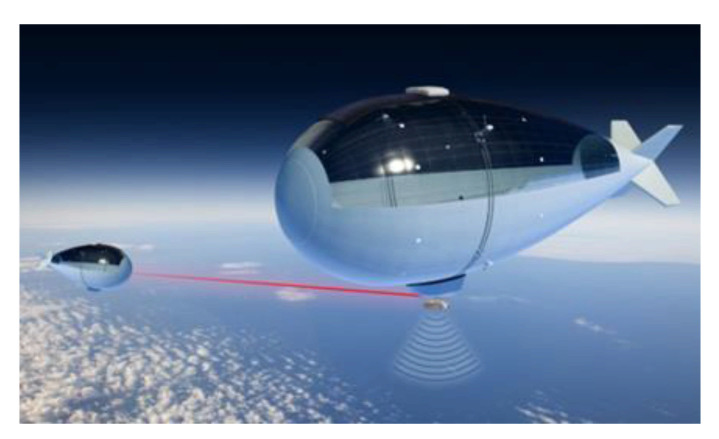
The Stratobus by Thalès [39].

Nevertheless, it is be a good starting point for the solution proposed later. In the same register as these flying telecommunication systems, we cite the Stratobus developed by the Thalès company in collaboration with other companies like MMIST in Canada [37], shown in Figure 6. It is an airship of 115 m long and 34 m in diameter that carries between 250 kg and 400 kg loads. It has flexible photovoltaic panels [38] on a section of its wing. A solar concentrator associated with a ring surrounding it allows the Stratobus to be permanently oriented towards the sun for better energy efficiency.

The airship by Thalès presents several options: it moves in the stratosphere [40] at an altitude of 20,000 m using electric motors and its autonomy in flight allows it to carry out missions of 5 years without maintenance. Moreover, there is no need for a launcher, and a control room on the ground enables its repositioning. It is planned to be deployed in 2025 to carry out land and sea surveillance. An intelligent system [37] will allow the photovoltaic panels integrated into its wing to be permanently facing the sun giving it flight autonomy of 5 years. Surveillance missions should quickly identify areas affected by natural disasters (land surveillance) and limit maritime piracy by strengthening the AIM identification system (maritime surveillance). Its deployment will also allow environmental management by monitoring the water’s cleanliness and the level of CO_2_ in the air. Its power in terms of the onboard telecommunication system will allow 5G communication. The project cost is estimated at 16.6 million euros, and the Stratobus can, if necessary, join the fleet of Loon balloons, although its power is infinitely greater.

#### 2.3.2. The Drones

**Figure 7:**
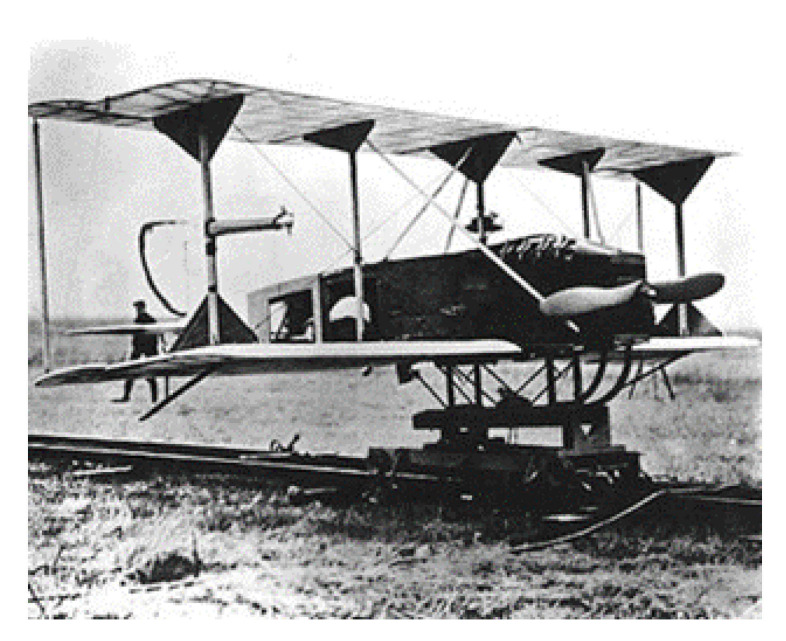
The world’s first drone developed by Archibald Low and team [41].

Drone history began in 1916 when the English engineer Archibald Low developed the aerial target (Figure 7), a crewless target plane, remotely piloted via radio waves. At the beginning of the 20th century, the great powers were looking for military applications to carry out air missions without putting their pilots’ lives at risk. The drone then underwent a considerable boom in the 21st century with recreational applications. Currently, the drone is a quadcopter with four brushless motors as a propulsion system, a chassis, and a speed controller.

**Figure 8:**
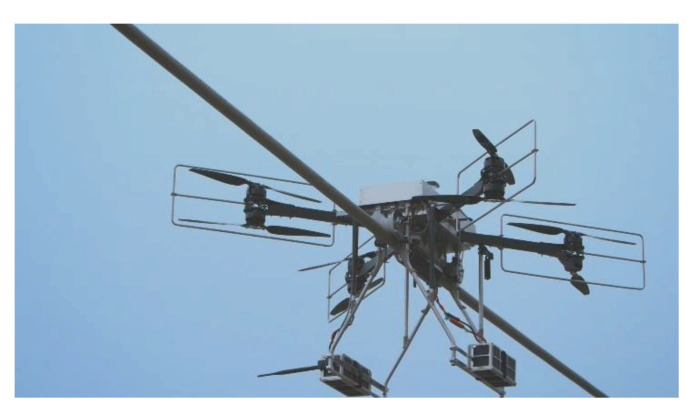
Hydro-Quebec’s line drone [43].

The size of drones ranges from a few tens of centimeters to a few meters for military applications. The small ones are used for leisure activities for individuals [42] by carrying a camera. Their weight is low, less than 250 g, and they do not require a specific permit in Canada, for example. From an industrial point of view, their field of applications is growing. Thus, they are used to assess future failures on structures such as bridges or pylons inaccessible to humans. In the same register of inspection, Hydro-Quebec is developing a drone called Line drone for the contact inspection of power lines, as in Figure 8.

**Figure 9:**
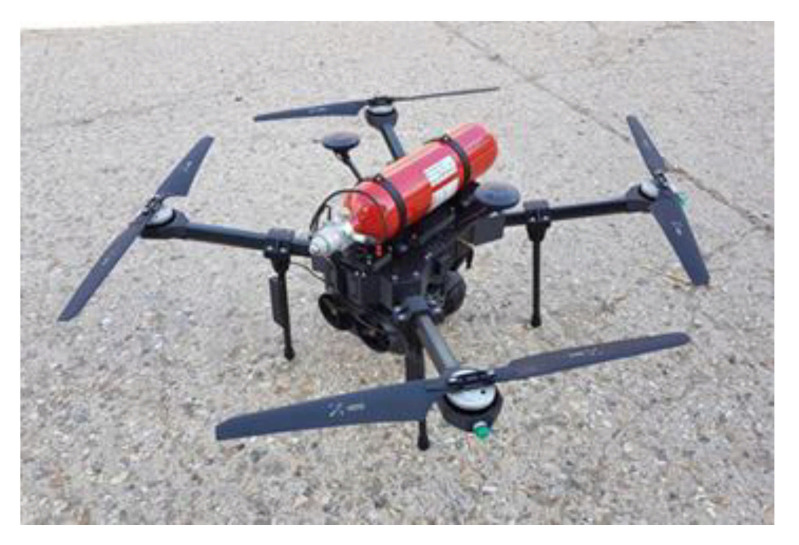
The Sensus drone [48].

The field of agriculture has integrated drones into its crop improvement processes or track animals [44]. Drones are indeed able to assess contamination in crop fields [45] with, of course, appropriate image processing. The drone field [46] is booming and many innovative aspects are being implemented. The British company ISS has unveiled its drone named Sensus (Figure 9). It is powered by a fuel cell [47] using hydrogen. Therefore, the drone takes on its hydrogen tank, thus giving it a flight autonomy of around 2 h. Different sensors can be installed to perform air analysis. A platform called Sensus has been developed to display and interpret the results of measurements made in flight.

**Figure 10:**
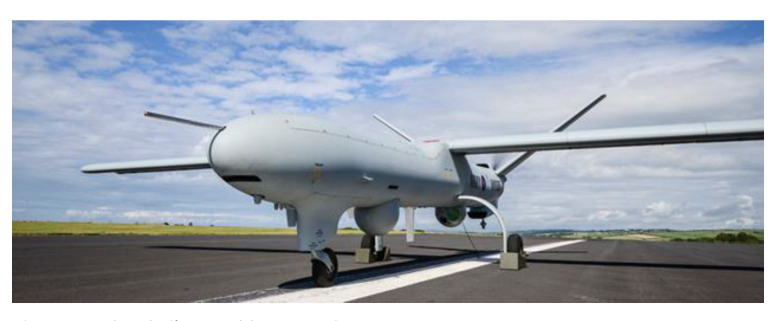
The Thalès Watchkeeper X drone [51].

Finally, to carry out operations without pilots, numerous drone applications supplement military drones [49]. The pilot room of these drones brings together trained military pilots. Thalès is developing giant drones like the watchkeeper X (Figure 10) to carry out surveillance missions. Its autonomy is 16 h and it is operational in less than 2 h. This autonomy allows it to intervene in places far from its base. These drones are crewless real scale planes. To ensure telecommunications coverage in an isolated sector, one could easily imagine a drone fleet, each drone carrying a telecommunication system flying over the area. This is like the Internet of drones (IOD) [50].

## 3. Comparison of the Solutions

### 3.1. Criteria for Comparing Solutions

We compared these different solutions and determined that they are adaptable to isolated and restricted conditions according to the following five parameters:

- The power supply of the telecommunication system and the autonomy of the system,
- The acclimatization of the telecommunication system to weather conditions,
- The range of coverage of the telecommunication system,
- The cost of the telecommunication system, and
- Telecommunication system deployment time and permanence.

#### 3.1.1. The Power Supply of the Telecommunication System and the Autonomy of the System

The telecommunication system that interests us is evolving in an isolated environment. One of the characteristics of this type of environment is the absence of an electrical network. The telecommunication system includes active structures to supply power for signal transmission, relay, or reception. Therefore, the electrical energy supply is a significant parameter for system characterization, a fortiori in an isolated environment. The use of renewable energies [52], photovoltaic or other, will be a preferred solution in the future. In this sense, the power supply of the telecommunication system is a significant criterion. In Northern Quebec, the energies widely used [53,54] are propane and diesel generators for electricity and fuel oil for direct heating. However, the energies mentioned above have high and increasing costs such as maintenance and fuel cost, increasing linearly for years, and transportation. The isolated environment [55] is an inaccessible place, and diesel transportation, for example, is costly. If we add these energies’ negative ecological impact, the energy supply systems in an isolated environment tend to be replaced with renewable energies. Industrial systems (telecommunication systems) in an isolated environment suffer the same effect. When it is a question of supplying systems that consume little electrical energy, photovoltaic panels (PV) [56] are preferred, as long as the annual sunshine of the isolated environment is sufficient [57]. They also offer great modularity.

We can indeed combine several in series to increase the voltage required to supply the loads or in parallel to increase the available current. PV technologies [58] are monocrystalline (16% to 24% efficiency) or polycrystalline (14% to 18% efficiency). If the system is more demanding, power can be supplied by wind turbines, which rated power can reach in the order of MWs. In this specific case, a study of the wind distribution (simulation and measurement campaign with a mast equipped with an anemometer on the site) will have to be carried out to justify the use of a wind turbine. It should be noted that the system should include batteries [59] to store the energy supplied by the wind turbine or PV and then supply loads. If the required electrical power is constant and extensive, hybrid solutions [60] are adopted. For example, they consist of renewable sources with a diesel generator [61], which takes over if required. The diesel generator starts when there is not enough wind in the case of a wind turbine or not enough sun in the case of PV and when the batteries are almost empty. An inverter must be added when the battery or the PV’s supply AC loads, as they are primarily connected to a CC bus.

The wind turbine usually provides AC used with a battery charger (AC-DC conversion) or a rectifier if needed to supply DC charges. All the energy flux in the hybrid system is managed by a controller that regulates the power flux between AC and DC buses, ensuring a balance between the power produced and consumed, using, as required, the battery storage. Numerous energy solutions for isolated environments [62] have emerged recently. Mobile solutions are available that integrate photovoltaic panels integrated on a structure, with wind turbines deployed during installation on site. The GreenCube solution [63] from the company ATI based in Rimouski, Quebec, is an example of this hybrid system type. The GC6 model can supply up to 6 kW of electrical power, sufficient for a remote telecommunication station. The deployment of these renewable energy solutions on isolated sites can be worth between a few hundred to hundreds of thousands of dollars depending on the solution chosen:

- PV only,
- Wind turbine(s) alone, or
- Hybrid systems (wind turbine, PV, diesel, and batteries).

**Figure 11:**
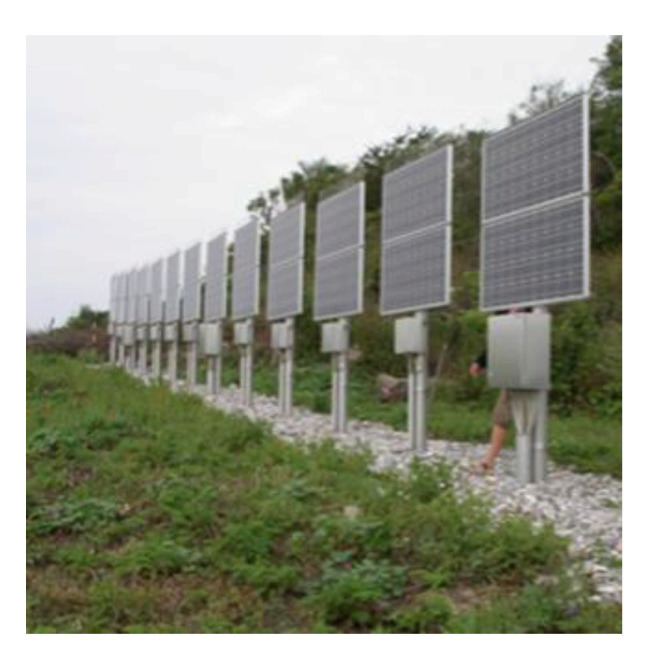
Vertically oriented PV [51].

To implement a telecommunication system in an isolated environment of the UHF type, for example, the powers involved in terms of system consumption (electronic cards) on the transmitter side as on the receiver side are very low. As an indication, the amplifier of a low-speed UHF transmitter using the ZigBee standard [64] requires only 1 W to get ranges of around 40 km with XBee XTEND modules. In the same vein, an Internet of Things system collecting sensor data on-site to transfer it in LoRa modulation [65] to the gateway works at 25 MW [66] in transmit power to reach transmitter-gateway distances of 22 km [67]. We chose the PVs to provide renewable energy in an isolated environment to power a telecommunication system alone [68]. To simplify installation, reduce costs, and mostly avoid ice and snow accumulation on panels, the PVs are often placed vertically, as shown in Figure 11.

Admittedly, the orientation is not optimal regarding the sun, but it avoids the deposit of frost or the accumulation of snow [69], which drastically reduces the PV return. Telecommunication towers, optical fiber, and flying telecommunication systems allow the use of photovoltaic panels to meet the telecommunication system’s needs in electrical energy.

For telecommunication towers and exit points or transmitters (relays) for optical fiber, photovoltaic panels are, most of the time, installed vertically. This limits the buildup of frost and prevents snow buildup. However, this solution is not optimal since the photovoltaic panel is not permanently oriented to face the sun.

The balloons overcome this drawback: the Stratobus by Thalès moves inside a ring that allows it to face the sun permanently. On the other hand, drones of conventional dimensions do not have photovoltaic panels since it is their onboard lithium-ion battery that supplies electrical energy. This is very limiting when you want to deploy a fleet of drones to ensure significant radio coverage. The flight autonomy due to the batteries is, on average, three hours, which practically condemns the use of drones for this type of mission.

#### 3.1.2. Adjustment of the Telecommunication System to Weather Conditions

**Figure 12:**
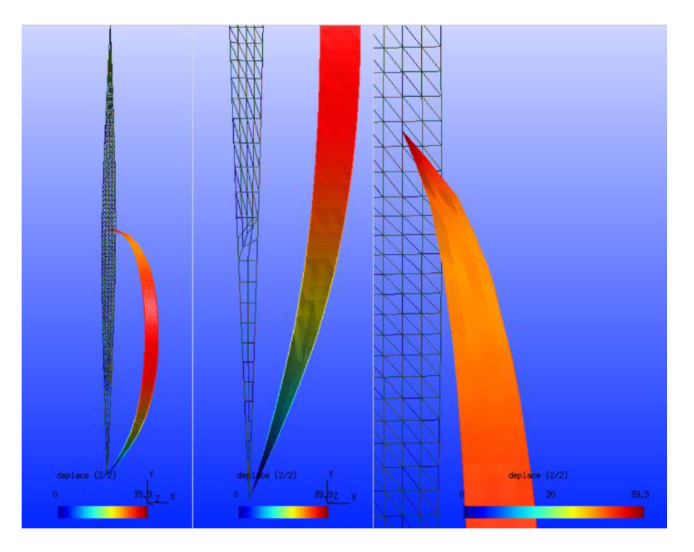
The spindles to make captive balloons.

The second criterion is closely linked to the weather conditions prevailing at the location of the telecommunication system. The system must indeed evolve in an environment constrained by the cold, which characterizes Northern Quebec. Therefore, the system has to be resistant to very low temperatures [70]. This constraint constitutes an important parameter because below −40 °C, the electronic devices’ operating limits are reached. It will be necessary, for example, to demonstrate expertise and, above all, a lot of experience to prevent the temperature from reaching this fateful threshold inside the cases (transmitters, receivers) containing the electronic devices. For example, solutions using heating wires should be implemented inside the casings. However, no solution is perfect. The heating system requires more energy from the system’s power supply. Another meteorological criterion that has an impact on the telecommunication system in an isolated environment is the wind. Indeed, the telecommunication system makes it possible to reach significant distances when the antennas are high up where the winds are the strongest. The signal’s reflections on the ground and obstacles, such as trees, are reduced at higher altitudes. The reflection of the signal transmitted on the ground (“ground reflection”) [71] results in a received power decay proportional to 1/*d*^4^ with respect to the transmitter–receiver distance. Therefore, the telecommunication system, at higher heights, faces strong wind resistance. Here again, the drones show their great weakness in deploying a telecommunication system. The strong wind is an enemy of the drone since it can nail it to the ground. Regarding the balloon solution for the deployment of a telecommunication system, the wind is also problematic. However, Loon balloons have been able to operate with winds of the order of 291 km/h in the stratosphere. The structure of the materials used to produce the balloon’s envelope [72] makes it possible to withstand excessively low temperatures. In general, a balloon’s envelope is built of several beams arranged like vertical strips [73]. All the beams (Figure 12) meet at the balloon’s north and south poles, thus constituting the balloon’s envelope. The spindles, just a few centimeters wide, consist of a complex of three thin films.

Most of the time, the first layer is polyethylene terephthalate (PET), about ten micrometers thick; the second layer consists of a polyamide 6.6 (PA) film twice the width of the first; and finally, a third layer consists of a PET film a few tens of micrometers thick. The spindles between the north and south points of convergence of the balloon are thermo-glued. The amount of helium injected into the balloon shortly before launch depends on the duration of the mission. The latter is intimately linked to the leakage of helium through the beams during the flight. For tethered balloons, if the photovoltaic structure is placed on the canopy, it would inevitably be covered for a more extended period than a fixed vertical orientation, as in the case of telecommunications towers.

On the other hand, telecommunication towers are a very good solution to resist strong winds and excessively low temperatures. Optical fiber is even more interesting, and it is the best solution since it is buried and, therefore, does not suffer the disadvantages of winds. At each point of the fiber where the access terminals or simply the repeaters are located, the photovoltaic entities for their power supply do not have dimensions that put them at risk in high winds.

#### 3.1.3. The Range of Coverage of the Telecommunication System

The third parameter concerns the coverage area of the telecommunication system. It depends on the propagation environment (geography and constitution of the atmosphere on the installation site), the carrier’s frequency, and the sensitivity of the receiver. The lower the sensitivity (minimum power of the received signal for proper restitution of information), the higher the transmitter-receiver distance. With the same aim of increasing the transmission distances and the covered area, the UHF waves (915 MHz) will be more suitable than a carrier frequency of 2.4 GHz or 5 GHz corresponding to Wi-Fi transmission [74]. On the other hand, the UHF frequencies do not make it possible to reach large data rates. For the sensor data transmission, limited in volume, UHF frequencies are very suitable. However, for video transmission, the Wi-Fi [75] is inevitably more convenient. In this specific case, the distance is limited. An alternative would be satellite transmission, the cost of which is excessive, especially in large volumes.

In comparing the different solutions according to the coverage criterion, it is necessary, as indicated above, to consider the height at which the antennas will be placed for telecommunication systems operating in UHF. The aerial solution with Loon or Stratobus balloons has an advantage as the balloons evolve in the stratosphere at altitudes of 20,000 km. The area covered [76] on Earth is, therefore, quite large. In any case, it is much higher than that achievable with telecommunication towers. According to legislation, drones can fly up to 90 m without special authorization, giving them an advantage compared to towers. Optical fiber is apart. Network coverage will depend on the kilometers of fiber deployed and the openings made in certain places to access the signal or send a signal. In any case, significant lengths of optical fibers will be required to cover the same surface as the balloons or telecommunication towers.

#### 3.1.4. The Cost of the Telecommunication System

The fourth criterion is economical. It simply consists of evaluating the operating cost of the system. This criterion is directly linked to the available budget for solution deployment. The first item of expenditure is the cost of manufacturing the telecommunication system associated with its deployment infrastructure. Loon and Stratobus balloons have relatively high manufacturing costs. It evokes sums in the order of 30 million Canadian dollars for the Stratobus. The structures of telecommunications towers are metallic or guyed with a moderate manufacturing cost of the order of a few tens of thousands Canadian dollars to a few hundred thousand Canadian dollars depending on the tower’s height. Optical fiber is relatively expensive at around 28,225 CAD per kilometer. The Éléonore mine’s connection to the network required the deployment of 124 km of optical fiber at the cost of 1.75 million CAD [77]. Indeed, it is necessary to dig trenches to bury optical fiber or use costly techniques to set up optical fiber underwater. Optical fiber is also very fragile, which requires many precautions and adequate equipment not only for its deployment. In fact, twenty-five million dollars have been spent since 2011 on the second portion of work for the installation of 800 km of optical fiber in Northern Quebec to connect nine Cree communities around James Bay (Canada). It should be noted that several lines were located 100 km from the James Bay road. Drones are, however, very interesting since we can cite the sum of a few thousand dollars for a drone.

The second expense item is the deployment of the system. The flying solutions stand out: balloons and drones take off without any specific methods. It is merely a matter of going to the take-off site to inflate the helium balloon, and it takes off alone. It is the same for drones, without the inflation operation. Telecommunication towers are deployed for amounts ranging from a few hundred thousand Canadian dollars to a million Canadian dollars. The elements of the metal structure must indeed be routed by helicopter in isolated, therefore challenging to access, places. This follows the work of preparation of the ground to clear the implementation area and make a flat and robust surface. This difficulty is reinforced when the soil is rocky, as in the case of Nunavik. We can cite two explicit examples of the cost a telecommunication tower. The Tshiuetin railway company had a telecommunication tower installed mainly for UHF antennas in Emeril (near Labrador city). The Emeril camp is accessible by road 500. This tower measures approximately 35 m. It was necessary to make a base to hold it. The cost of this telecommunications tower was 200,000 CAD. The material for building this tower was transported by road. The second example is still in the state of a call for tender. It was always a question of installing a telecommunication tower in Faden near Schefferville on the Tshiuetin company’s railway. The tower is 70 m in height. Its cost is around one million dollars, given that there is no road access to Faden and that a helicopter is required to transport the metal structure and complete the installation. As for the optical fiber, its installation requires digging trenches along its future course. Depending on the nature of the soils, rocky or not, and on the difficulty of access to the land (isolated environment), this can take time and significant human investment. Likewise, generally, the fiber is implanted in pieces that are joined together to achieve the desired total length. The technique for making these junctions is unique. These junctions must be flawlessly performed to limit signal losses. This requires special equipment and specialists. The installation of optical fiber to cover an area is therefore very expensive.

The last item of expenditure is the cost of operating the telecommunication system; in other words, maintenance costs. The leading solution is an optical fiber. Once deployed, its reliability is high and human intervention is very limited unless a repeater breaks, for example. Breakings occur very rarely because the optical fiber is underground. Furthermore, telecommunication towers do not require significant interventions except possibly to repoint the antennas. In this specific case, human intervention on large towers is delicate. Therefore, the intervention cost can be high if it necessitates the use of a helicopter or the transportation of the intervention team. The balloons seem to be a good alternative even if these solutions are emerging and it is too early to assess their reliability. However, if the strong winds do not alter the balloon’s structure, the only periodic intervention is to recover the balloon, re-inflate it, and send it back. As for the drone, it requires an important intervention cost, especially as a permanent solution. The flight time is on average 3 h; this requires untimely recovery of the drone, recharging the batteries, and probably repairing the drone’s light elements if the weather conditions are harsh.

#### 3.1.5. Telecommunication System Deployment Time and Permanence

**Figure 13:**
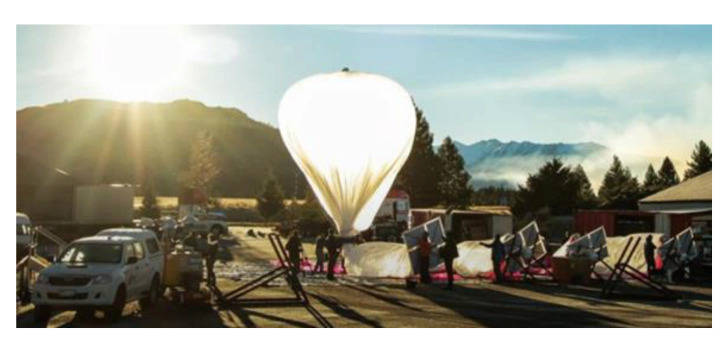
Deployment of Google Loon before launching [78].

The last parameter to evaluate the best solution is whether the system deployed can be operated on an occasional or permanent basis and deployment time. The occasional solution can be interesting when, for example, a natural accident of an environmental type occurs in an isolated region. The establishment of an occasional telecommunication system will make it possible to locate individuals, possibly in danger. An LTE network provides a vital connection to organize help. The case of the hurricane that swept through Puerto Rico in October 2017 is an example of the contribution of an occasional telecommunication system. The Loon balloon fleet very quickly deployed [77] on-site during this tragic event made it possible to restore the network to some people in distress. They could then send an SMS or an email, be located, and then be saved. The balloons provide a permanent solution when the materials used to make the envelope limit the helium leakage over time. Then, the balloons must land to be re-inflated only after an extended period, thus giving the system permanence on site. This is the case for Loon balloons or for the Stratobus. Regarding the deployment time, it is relatively low and well established, as illustrated in Figure 13.

The balloon is brought to the launch site, inflated, and allowed to take off. Depending on the launch location chosen, this time will be the team’s traveling time, the preparation time of the equipment, which is on average two days, a day for the launch, and the team’s return. A week-long campaign is usually enough. For the drones, it is merely a matter of sending the team in situ. On the other hand, the deployment time of a telecommunications tower is longer. We often talk about a few weeks depending on the geography of the implementation site in an isolated environment. The slowest solution to deploy is an optical fiber. The ground must first be prepared (brush cutting, possibly blasting, etc.) and trenches made, over distances which can be significant. It is then necessary to install the “ends of optical fibers” and carry out the junctions. It can take months. Optical fiber installation took four months between Emeril and Schefferville in Northern Quebec. It is a typical isolated environment, and the fiber was deployed over more than 250 km along the railroad operated by the Tshiuetin railway company. It is, therefore, the most prolonged solution to implement.

## 4. Assessment of Solutions Comparisons

As presented in Section 3, each of the solutions mentioned above has advantages and limitations. We compared in Table 2 the different solutions according to the five criteria discussed above. The comparison uses a normative assessment from A (as the best solution) to E (as the worst solution).

The table shows the dominance of the flying solution and, more particularly, of the balloons. However, such a solution’s limiting factor can be the cost with major architectures like the Stratobus. However, a solution with smaller tethered balloons (connected to the ground) that does not require a ground control room to pilot them and large teams for their design and development would be better adapted to the isolated and constrained environment.

## 5. The Tethered Balloon Solution

### 5.1. Description of the Captive Balloon

**Figure 14:**
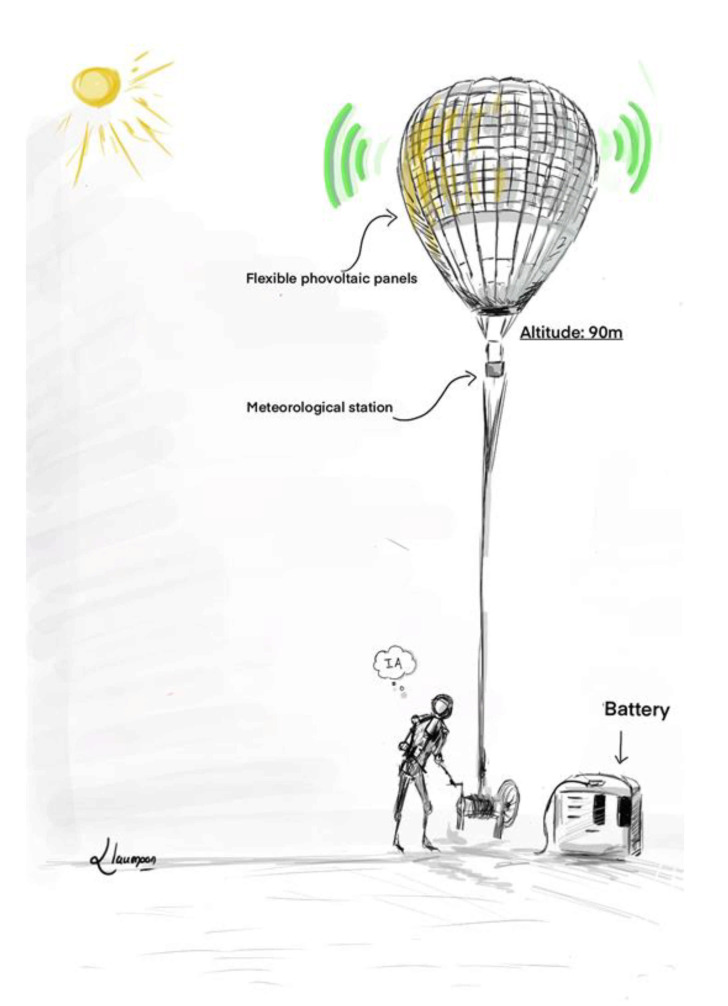
Illustration of the telecommunication balloon.

**Figure 15:**
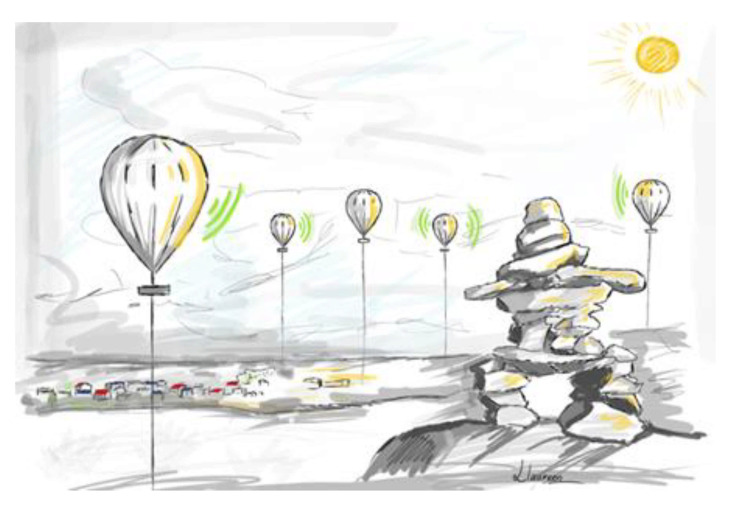
Illustration of the telecommunication coverage for an isolated sector with captive balloons.

The comparative study shows that a flying solution based on captive tethered balloons [79] can be the best to implement a telecommunication system in an isolated and constrained environment like Northern Quebec. The solutions developed by Google (Google Loon) and Thalès (Stratobus) require significant human resources with teams managing the balloon’s development, teams taking charge of the launch, and finally, a team monitoring the flight of the balloon in a control room. Under these conditions, the cost of such a solution quickly becomes an obstacle when one wishes to implement a telecommunication system in a localized environment and with reduced budgets compared to the millions of dollars spent by Google and Thalès. These solutions are so attractive in terms of radio coverage and in terms of energy autonomy, all associated with good performance; therefore, a more modest solution with captive balloons becomes very interesting. The tethered balloon (Figure 14) has a “wire at its leg” linking it to the ground. Photovoltaic panels fit the balloon envelope. Thus, the solar energy captured is converted into electrical energy and sent to the ground to charge the batteries that supply the balloon’s onboard telecommunication system. An intelligent control system ensures the balloon’s stability in flight during strong wind conditions. The balloon’s height can be around 90 m depending on the results obtained in the simulation (RadioMobile software) to size the telecommunication system. A fleet of balloons is deployed to obtain telecommunication coverage for an isolated sector. The balloons will communicate with each other thanks to their onboard telecommunication system (Figure 15). If one of the balloons is connected to the Internet by optical fiber, then all the balloons have the Internet. We can, therefore, imagine disseminating sensors [80] on the isolated site.

This telecommunication structure will initially allow data from sensors located in the isolated sector to be pushed into the “Cloud” [81], thus enabling remote monitoring of the isolated sector accompanied by SMS or email alerts if necessary. The sensors can be for environmental applications to prevent a climatic disaster or allow the geolocation of individuals who might need assistance. In the same vein, this monitoring could apply to industrial processes on railroads or in mines. Then, in the case of trains, it would be possible to follow at a distance the trains operating in isolated areas and for mining companies to remotely monitor their industrial processes. Therefore, we will have deployed the Internet of Things [82,83] in an isolated environment. This solution has the advantage of being mobile because it can be deployed in a short time.

**Figure 16:**
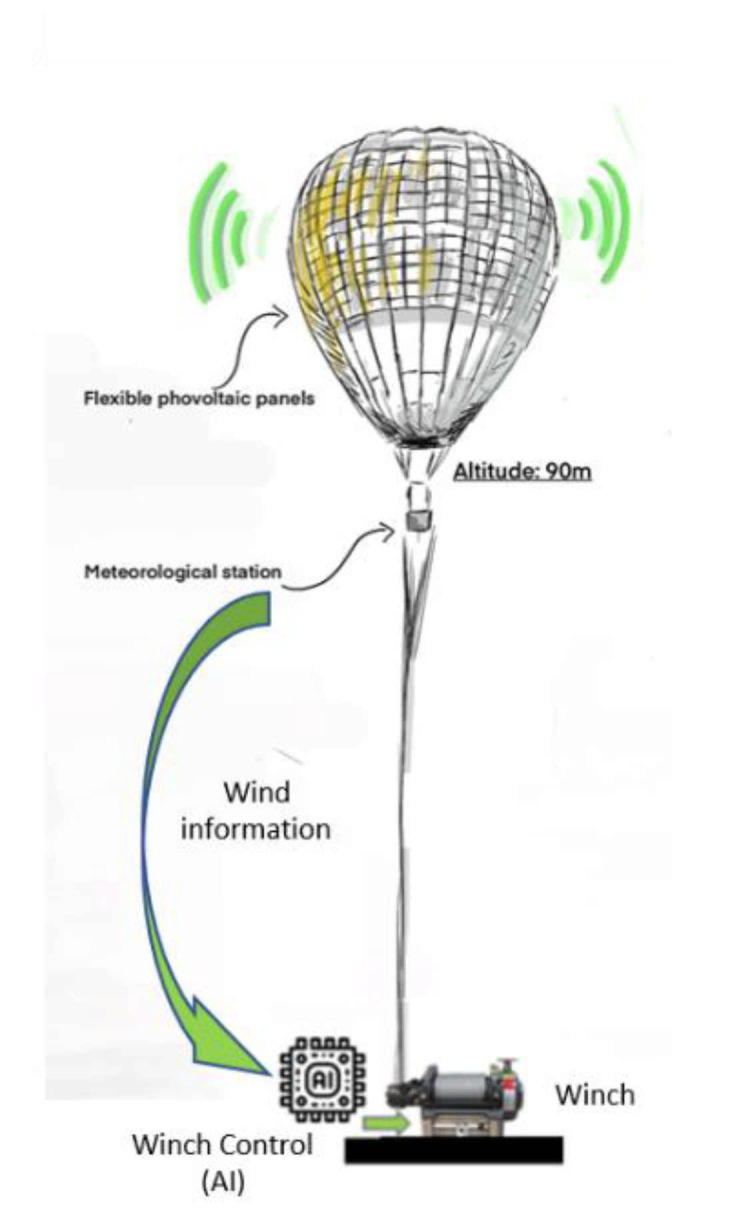
Artificially intelligent control of the balloon.

However, to withstand strong winds, the cables connecting the balloon to the ground will be controlled by an intelligent control system, fed by data from a mini meteorological station embarked on the balloon, as in Figure 16.

### 5.2. Balloon Technology: The Physical Structure

From a technological point of view, these captive balloons will be inflated with helium. Their external envelope will be close to that of the Loon balloon: three PET-PA-PET layers will constitute the balloon’s thickness to form beams. The balloon’s final structure will be the beams’ junction converging on its top and its base. Four cables spaced 90° apart and attached to the balloon will roll on a winch system on the ground. This system will deploy the balloon and, of course, lower it back to the ground.

### 5.3. The Telecommunication System

**Figure 17:**
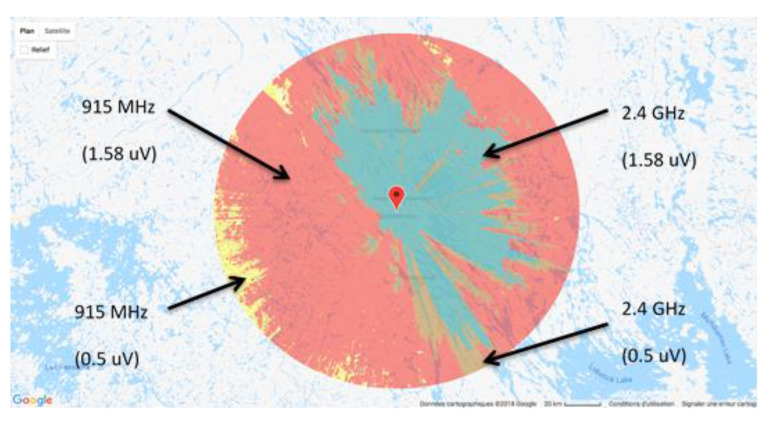
Illustration of the Schefferville radio coverage.

Simulations of radio coverage performed upstream will define the flight height. In this register, software like mobile radio can be handy. By fixing the height of the emission source, the transmitter’s power, the receiver’s sensitivity, and the losses in the transmission chain, it allows the sector covered to be represented [84]. It uses the Longley Rice model, which considers the geography and the composition of the site’s atmosphere to be assessed. Figure 17 shows a radio coverage study on the Schefferville site (Northern Quebec) with a height for the transmitting antenna of 90 m and transmitter power of 1 watt corresponding to XBee Pro modules. Two frequencies were studied, 915 MHz and 2.4 GHz, for each of the two different sensitivities (0.5μV and 1.58 μV) for the receiver. We note that the 915 MHz gives broader coverage with the 0.5 μV sensitivity. The area covered is the complete disc.

### 5.4. Ecological Energy Supply

**Figure 18:**
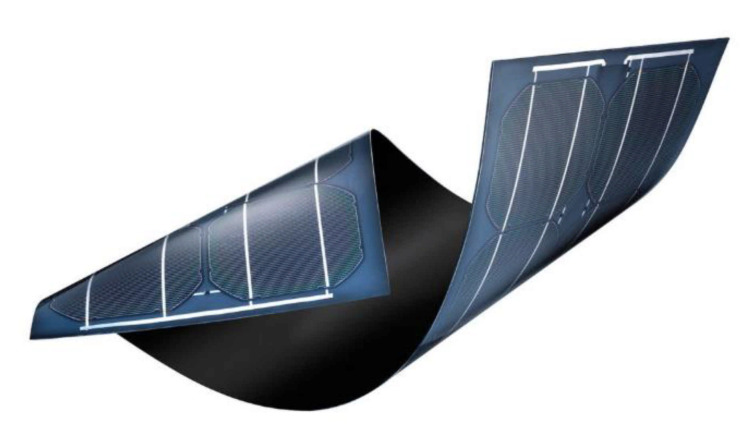
Ultra-thin and flexible photovoltaic panels [86].

As seen in Figure 18, flexible photovoltaic panels will achieve the autonomy of the balloons in electrical energy. This technology underwent a major boom when a research project carried out in 2012 made it possible to create thin-film photovoltaic cells a few micrometers thick. Xiaolin Zheng carried out this study at Stanford University in the United States [85]. Thanks to their small thickness, these structures have the advantage of being flexible and light. However, they must be built on the same substrate, which was the study’s main problem.

Nevertheless, the scientific team managed to create a reusable substrate, which was very complicated because the substrate had to, among other things, have a homogeneous surface, resist very high temperatures, and not react with certain chemicals used in the manufacturing of photoelectric cells. The solution was to make the thin film photoelectric cells on a rigid substrate consisting of a wafer of silicon dioxide (SiO_2_) on which was deposited a layer of nickel 300 nm thick. Then, the different layers constituting the current producing unit were deposited conventionally. As part of the proof of concept, the deposited layers were made of hydrogenated amorphous silicon.

The layers were then covered with a protective polymer on which was fixed a transparent and heat conductive transfer sheet used to transport the cell. In a second step, the still-rigid product elements were immersed in water at room temperature. Slight traction was exerted on the transfer sheet allowing the water to penetrate between the wafer and the nickel until the separation of these two layers. The cells were then extracted from the water and heated to 90 °C to make them flexible. They could then be pasted anywhere (telephone, electronic business card) using double-sided adhesive or glue. After, the sheet was removed, and the silicon wafers could be reused to manufacture new cells. The conversion efficiency obtained during this experiment remains unchanged after the transfer. It was 7.7% on the rigid substrate and remained identical after transfer. Using other types of cells (CIGS) composed of copper, indium, gallium, and selenium, the efficiency achieved was 22.6%. The photovoltaic panels’ arrangement on the balloon envelope will optimize their performance because as the sun makes its way around, the photovoltaic cells capture the sunlight all day. This solution is deployable very quickly. It suffices to carry the balloons on-site to set up the ground-fixing system and the batteries and then launch the balloon. The balloon is operational 24 h a day. Depending on its size, it can carry loads of up to 250 kg. Its operating cost is less than 100 CAD per hour. The side that will require improvement is flight autonomy. Currently, captive balloons with this type of structure (PET-PA-PET) and the inherent helium leaks last approximately ten days in the air. It is then necessary to readjust the volume of helium. The fleet of balloons deployed on-site can respond to occasional problems such as during a sudden environmental event when communications are broken and there is no longer any energy supply, or when one wishes to carry out a measurement campaign over an isolated and vast territory.

On the other hand, if a new combination of materials makes it possible to increase the flight time to one month, the fleet of tethered balloons constituting the telecommunication system in an isolated and constrained environment may be used permanently. It would not be absurd to send a person, once a month, equipped with helium refills in a remote environment to re-inflate the balloons. From then on, it would be possible to follow trains carrying passengers or iron ore, as the Tshiuetin railroad company in Northern Quebec does, and all the company’s rolling stock. This would provide significant additional safety. Likewise, mining companies operating in Northern Quebec could monitor their production sites remotely.

## 6. Conclusions

Many vast territories within Canada, Russia, China, and India are isolated regions and are disconnected from the rest of the world. Today, it is essential to establish adequate telecommunication systems in these places, namely, reliable and fast enough techniques to reconnect them to the rest of the world. For now, the only solution is the satellite, but it comes at a very high cost. Therefore, the future will have to offer affordable, green, and reliable solutions. As part of this article, a state of the art of the solutions that can be implanted in an isolated environment was conducted with a critical analysis based on several criteria. This study made it possible to identify a suitable solution: the captive balloon. This solution has many advantages for these isolated regions, but it also presents challenges requiring research work. The materials to make the envelope of the captive balloon already exist, but it will be necessary to make sure that the photovoltaic panels installed on the airfoil do not modify the characteristics of the textile used, such as:

- Its resistance to cold and
- Its ability to retain gas, which will have a direct impact on flight autonomy.

Safety is also an item requiring further study. It will be necessary to ensure that the balloon does not exceed 120 m in height, a height beyond which authorizations are required from Transport Canada. Similarly, the balloon’s stabilization in flight facing the wind will require developing an innovative, intelligent control system with, possibly, an artificial intelligence layer. Finally, powering the system over a large area could pose problems with different weather conditions from one balloon to another. The choice of a telecommunication system that provides the greatest range while consuming the least possible energy is also a challenge. It will also have to be at the current standard and follow the evolution of technology, which is very fast in this sector. Without this, the disconnection of isolated environments will persist.

## Figures and Tables

**Table 1:** Comparison of the different WDM multiplexing.

	Coarse WDM	Dense WDM	Ultra-Dense WDM
Number of wavelengths	<17	8 to 128	>400
Channel spacing	20 nm to 25 nm	0.4 nm to 1.6 nm	0.08 nm
Spectral width	1260 nm to 1620 nm	1500 nm to 1600 nm	1500 nm to 1600 nm
Flow by wavelength	1.25 to 2.5 Gbits/s	10 Gbits/s to 40 Gbits/s	>40 Gbits/s

**Table 2:** Summary comparison of telecommunication system solutions in isolated areas and constraint environment (A is the best solution, and E is the worst solution).

	Electrical Supply	Acclimatization Depending on Weather Conditions	Radio Coverage	Cost of the System	Deployment Time
Telecommunication towers	C	B	B	D	D
Optical fiber	C	A	C	E	E
The balloons	A	C	A	B	B
The drones	D	E	B	B	A

## Data Availability

This study does not report any data.
