# Correction: Ferrier et al. State of the Art of Telecommunication Systems in Isolated and Constrained Areas. *Sensors* 2021, *21*, 3073

**DOI:** 10.3390/s26102941

**Published:** 2026-05-08

**Authors:** Laurent Ferrier, Hussein Ibrahim, Mohamad Issa, Adrian Ilinca

**Affiliations:** 1 Wind Energy Research Laboratory, Université du Québec à Rimouski, Rimouski, QC G5L 3A1, Canada; adrian_ilinca@uqar.ca; 2 Institut Technologique de la Maintenance Industrielle, Sept-Îles, QC G5L 3A1, Canada; hussein.ibrahim@itmi.ca; 3 Institut Maritime du Québec à Rimouski, Rimouski, QC G5L 3A1, Canada; missa@imq.qc.ca

**Text Correction**

In the original publication [1], there are several unclear expressions. The corrections are as follows:

In the Abstract, the sentence “Furthermore, the magnetic field in some regions of the planet and very often in isolated areas undergoes partial or total absorption, known as white areas, making the propagation of the signal very delicate” should be replaced by “Furthermore, in many remote regions of the planet, radio communication remains difficult due to geographical constraints, environmental attenuation, and the lack of telecommunication infrastructure. These regions are often referred to as “white areas,” meaning zones with little or no communication coverage”.

In the eighth paragraph of the Introduction, the sentence “The magnetic field in some regions of the planet and very often in remote areas undergoes partial or total absorption known as white areas [11], making the propagation of the signal very delicate [12]” should be replaced by “In many remote regions of the planet, radio communication remains difficult due to geographical constraints, environmental attenuation, and the lack of telecommunication infrastructure. These regions are often referred to as “white areas [11],” meaning zones with little or no communication coverage [12]”.

In the first paragraph of Section 3.1.2, the sentence “The reflection of the signal transmitted on the ground (“ground reflection”) [71] generates a division by 4 of the distance of the couple transmitter-receiver in the transmission of Hertzian information” should be replaced by “The reflection of the signal transmitted on the ground (“ground reflection”) [71] results in a received power decay proportional to 1/*d*^4^ with respect to the transmitter–receiver distance”.

The authors state that the scientific conclusions are unaffected. This correction was approved by the Academic Editor. The original publication has also been updated.
